# A comprehensive analysis on nanostructured materials in a thermoelectric micro-system based on geometric shape, segmentation structure and load resistance

**DOI:** 10.1038/s41598-020-78770-9

**Published:** 2020-12-10

**Authors:** Miguel Angel Olivares-Robles, Carlos Alberto Badillo-Ruiz, Pablo Eduardo Ruiz-Ortega

**Affiliations:** 1 Instituto Politécnico Nacional, SEPI, Escuela Superior de Ingeniería Mecánica y Eléctrica Unidad Culhuacan, 04430 Coyoacan, Mexico City, Mexico; 2 Instituto Politécnico Nacional, SEPI, Escuela Nacional de Ciencias Biológicas, 11340 Mexico City, Mexico

**Keywords:** Devices for energy harvesting, Thermoelectric devices and materials

## Abstract

In this study, we report the novel energy behavior of high-performance nanostructured materials in a segmented thermoelectric micro-generator (TEG). Several physical elements of the materials must be considered to determine their behavior in the thermoelectric energy conversion: temperature dependence of material properties, geometric structure, segmentation, and the symmetry of each or both p-type and n-type nanostructure semiconductor thermoelements. Recently, many efforts have reported effects independent on the thermoelectric performance of semiconductor materials. In this work, exhaustive research on the performance of high-performance nanostructured materials in a segmented thermoelectric micro-generator (TEG) was carried out. Our results show the efficiency and output power of the TEG using the temperature-dependent model, i.e., a variable internal resistance for a load resistance of the system. Our approach allows us to analyze symmetrical and asymmetric geometries, showing maximum and minimum peaks values in the performance of the TEG for specific $\gamma$ values. The performance of the TEG is improved by about $6\%$ and $7\%$, for efficiency, and output power, respectively, considering a trapezoidal geometric shape in the 2p-3n segmented system, compared with the conventional rectangular shape.

## Introduction

A thermoelectric generator (TEG) is a solid-state device with significant advantages, no moving parts, no constant maintenance, not use fossil fuels and silent in operation, etc. In recent years the benefits of TEG have caught interest as a viable alternative in generating energy friendly to the environment. However, the efficiency of commercial thermoelectric devices does not exceed 5–10$\%$^1,2^. There are three methods to improve the performance of thermoelectric systems: material preparation, device design, and operation control. The development of new materials seeks to obtain materials with a high figure of merit values. The device’s design requires an optimal relationship between the geometric factors, length, and cross-sectional area of the thermoelements, while the operating conditions of the TEG analyze the difference in temperature, thermal and electrical resistance, among other factors^3^. Recent researches have shown novel methods to improve the efficiency of thermoelectric materials; in general, nanostructuring is focused on reducing thermal conductivity without significantly affecting electrical conductivity and increasing the Seebeck coefficient. Nanostructured materials have been an important advance in the optimization of the figure of merit (ZT). The maximum efficiency of a thermoelectric material depends on thermoelectric properties through the figure of merit, $ZT=\alpha ^2\sigma T/\kappa$, where $\alpha$ is the Seebeck coefficient, $\kappa$ is the thermal conductivity, and $\sigma$ is the electrical conductivity. It is well known that nanostructured materials can exhibit higher figures of merit values than bulk materials, even in high temperatures ranges. For example, the bulk material $Bi_2Te_3$ has a merit figure of $ZT=0.7$, while the nanostructured material 2.5$\%$ K-doped $PbTe_{0.7}S_{0.3}$ shows a figure of merit $ZT>2$^4,5^. Hu et al.^6^ proposed a method based on hot deformation (HD)-induced multi-scale microstructures. They showed that the thermal conductivity of the lattice was considerably reduced by the presence of in situ nanostructures, which were induced by recrystallization and high-density lattice defects. The preferred orientation formation during the HD process improves the electrical conductivity, and the donor effect partially compensates for the concentration of the gap, increasing the Seebeck coefficient. The high performance of nanostructured materials is due to the reduction of bipolar conduction in thermal conductivity through the nano inclusions by milling and spark plasma sintering^7^. Furthermore, it has been proved that by controlling hierarchical architecture, from atomic-scale to mesoscale of a PbTe p-type nanostructured material, a large enhancement in the thermoelectric performance is achieved with values of $ZT>2$. Thereby, multi-scale hierarchical architecture in controlling phonon scattering offers a realistic prospect of the recovery of a significant portion of waste heat^8^. Determine the optimal geometric parameters, i.e., cross-sectional area, length, and the number of thermoelements pairs, which directly affect the performance of TEG systems is another strategy to optimize energy harvesting from waste heat. Recent work showed that changing the shape of the legs (trapezoidal, rectangular, hexagonal, and cylindrical) and the alignment of the elements (inclination angle between the edge and the base), impacts temperature distribution, output power, efficiency conversion, and thermal stress distribution and also can improve TEG performance^9–11^. Shittu et al.^12^ showed that when making a severe geometric modification in one of the thermoelements, the output power can be increased; In the case of a rectangular thermoelement in conjunction with a trapezoidal thermoelement, the effect of asymmetry and segmentation is reflected in the increase of the output power by 117$\%$ in addition to reducing the thermal stress. However, it should be noted that the increase in output power is compared with a conventional TEG, so in this work, we analyze the geometric effect of asymmetry and segmentation with equivalent systems.

Ouyang et al.^13^ show a segmented thermoelectric system with symmetric and asymmetric models. Symmetrical models are constructed with an area of 1474.56 mm^2^, it combines traditional and nanostructured thermoelectric materials with the constant load resistance. The optimization of the area relationship between thermoelements ($A_n/A_p$), number, and thickness of the thermoelement. The results show that higher output power densities are feasible in an optimal geometric relationship with $\Delta$T = 500–700 K. It also shows that an asymmetric geometry is necessary to obtain the optimized TEG performance.

Recent investigations of annular thermoelectric generators (ATEG) and segmented annular generators (SATEG) show that the performance is optimal when the annular shape parameter $Sr=1$, that is, the power increases with the increase of *Sr* , reaches its maximum value when it becomes a flat-plate thermoelectric and decreases the power again. It has also been shown that to improve the heat transfer capacity and efficiency, the contact area of the high temperature zone ($T_h$) can be increased^14–16^.

Ali et al.^17,18^ studies the influence pin shape configuration and with exponential area variation of the thermoelements on the performance of segmented TEGs. It shows that, the proper selection of the geometric shape of the thermoelement, temperature ratio ($\theta$), and external load parameter ($R_L/R_0$) results in an improvement in the performance of the thermoelectric generator in terms of efficiency and power output compared to the single material pin configuration.

The literature review performed for segmented systems with the influence of geometry on TEG performance shows the vital importance of improving methods to optimize TEG. Therefore, a simultaneous analysis is performed, using: Segmented TEGs with different segmentations, the evolution of the geometric shape parameter $\gamma$, symmetric and asymmetric thermoelements, different temperature gradients, temperature-dependent high-performance nanostructured materials, internal and load resistance constant, and variable, to determine the best design selection.

In this study, a TEG with asymmetrical and segmented legs of three different thermoelectric nanostructured materials is analyzed. As it is well known, p-type and n-type legs have traditionally uniform cross-section areas, i.e., rectangular geometries. Here, in this analysis, we proposed an asymmetrical model with complex geometries that will help understand the effect of the non- uniform geometry, defined as the geometric factor $\gamma$, on the performance of the system to make it more feasible for applications. This work is organized as follows. “Method” section provides our proposed model of a TEG with the equations that govern the system, a description of thermoelectric properties of the temperature-dependent materials, the different geometric models, and the configurations of the materials. The results and discussion of the effect of the geometric factor and load resistance on efficiency and output power are presented in “Results and discussion” section, and the relevant conclusions of this study are in “Conclusion” section.

## Method

### System modeling and governing equations

In this paper we we make use of the irreversible thermodynamics principles for energy conversion which are the basis of thermoelectricity. Onsager’s linear theory describes the interaction of heat and electric current fluxes in a thermoelectric process through the kinetic coefficients which obey Onsager’s reciprocity relations^19,20^. The heat flow equation considering the endothermic processes and the exothermic Peltier effect is given by

1 $$\begin{aligned} \nabla \cdot \left( \kappa \nabla T\right) -T{\mathbf{J}}\cdot \nabla \alpha =\frac{-J^2}{\sigma } \end{aligned}$$

where $\kappa$, ${\mathbf{J}}$, $\alpha$, and $\sigma$ are the thermal conductivity, current density, Seebeck coefficient, and electrical conductivity, respectively. In general, $\kappa (T)$, $\sigma (T)$ and $\alpha (T)$ are dependent on the temperature. Considering the Seebeck effect, the equation of the electric field is given by

2 $$\begin{aligned} \nabla \cdot \left( \sigma \nabla V+\alpha \sigma \nabla T\right) =0 \end{aligned}$$

The electric potential is defined as *V*. The Peltier effect, which explains the heat flux **q** in the thermoelements, is expressed by

3 $$\begin{aligned} {\mathbf{q}}=\kappa \nabla T + \alpha {\mathbf{J}}T \end{aligned}$$

The differential equations are solved simultaneously. The solution of the simultaneous Eqs. (1)–(2) is obtained with the finite element method in a three-dimensional space. Equations (1)–(3) are combined to get the heat transfer rate of the cold side ($Q_c$) and the heat transfer rate of the hot side ($Q_h$) of the TEG.

The output power $(P_e)$ of the system can be expressed in terms of load resistance $R_L$

4 $$\begin{aligned} P_e=I^2R_L \end{aligned}$$

The voltage is defined as $V=\alpha \left( T_h -T_c\right)$ and the electric current is expressed as

5 $$\begin{aligned} I=\frac{V}{R_L+R_{int}}=\frac{\alpha \left( T_h -T_c\right) }{R_L+R_{int}} \end{aligned}$$

where $R_{int}$ is the internal resistance of the TEG. The internal resistance of the thermocouple $R_{int}$ can be written more explicitly as

6 $$\begin{aligned} R_{int}=\frac{1}{A_{p}}\left( \frac{L_{1}}{\sigma _{p1}}+\frac{L_{2}}{\sigma _{p2}}+\frac{L_{3}}{\sigma _{p3}}\right) +\frac{1}{A_{n}}\left( \frac{L_{1}}{\sigma _{n1}}+\frac{L_{2}}{\sigma _{n2}}\right) +R_{cu} \end{aligned}$$

where subscript 1, 2, 3 are Segment 1, Segment 2, and Segment 3, respectively. The index *p* or *n* refers to the type of material, and $R_{cu}$ is the resistance of copper electrodes. The relationship between the load resistance and the internal resistance of the TEG defined as

7 $$\begin{aligned} m=R_L/R_{int} \end{aligned}$$

The efficiency of the thermoelement $\eta$ is given by

8 $$\begin{aligned} \eta =\frac{P_e}{Q_h}=1-\frac{Q_c}{Q_h} \end{aligned}$$

**Figure 1:**
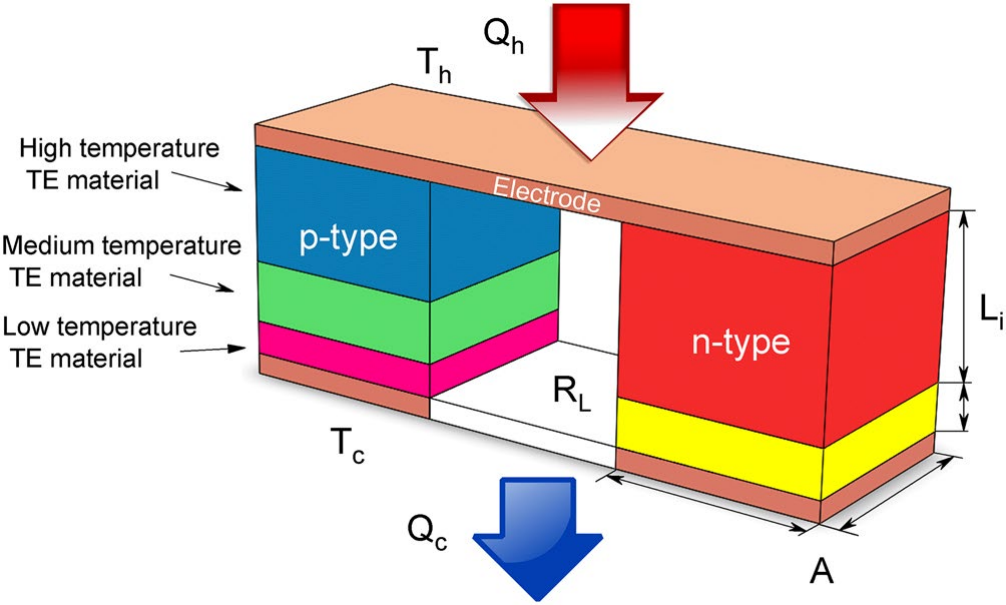
Micro segmented thermoelectric generator (TEG).

The segmented TEG model is composed of p-type and one n-type thermocouple, three copper electrodes, and load resistance, which are electrically connected in series and thermally in parallel, as shown in Fig. 1.

Where $L_i$ is the length of each segment, and *A* is the cross-sectional area for the p-type or n-type thermoelement. In the temperature difference $\Delta T=T_h-T_c$, we set $T_c=300$ K and $T_h$ is the range of 310 to 900 K. To show the effect of a geometric factor on the performance of the TEG, we define a parameter $\gamma$ that relates the cross-sectional area of the thermoelements,

9 $$\begin{aligned} \gamma =A_H/A_L \end{aligned}$$

where $A_L$ and $A_H$ are the cross-sectional areas of the heat rate of the cold side and the hot side of the thermoelement, respectively. TEG’s has been characterized by having a low performance due to the use of conventional materials that only allow operating below 200 $^{\circ }$C. Recently has been shown that using techniques such as nano-structuring in thermoelectric materials, TEG’s can operate at temperatures above 500 $^{\circ }$C, making TEG devices now a commercially viable technology^21^. The structures analyzed in this paper have been studied in the literature considering governing equations here presented but with the different optimization approaches. Here, we give an understanding of the thermal and electrical properties of heterogeneous structures in order to improve design and efficiency.

### Temperature dependent nanostructured material properties

**Figure 2:**
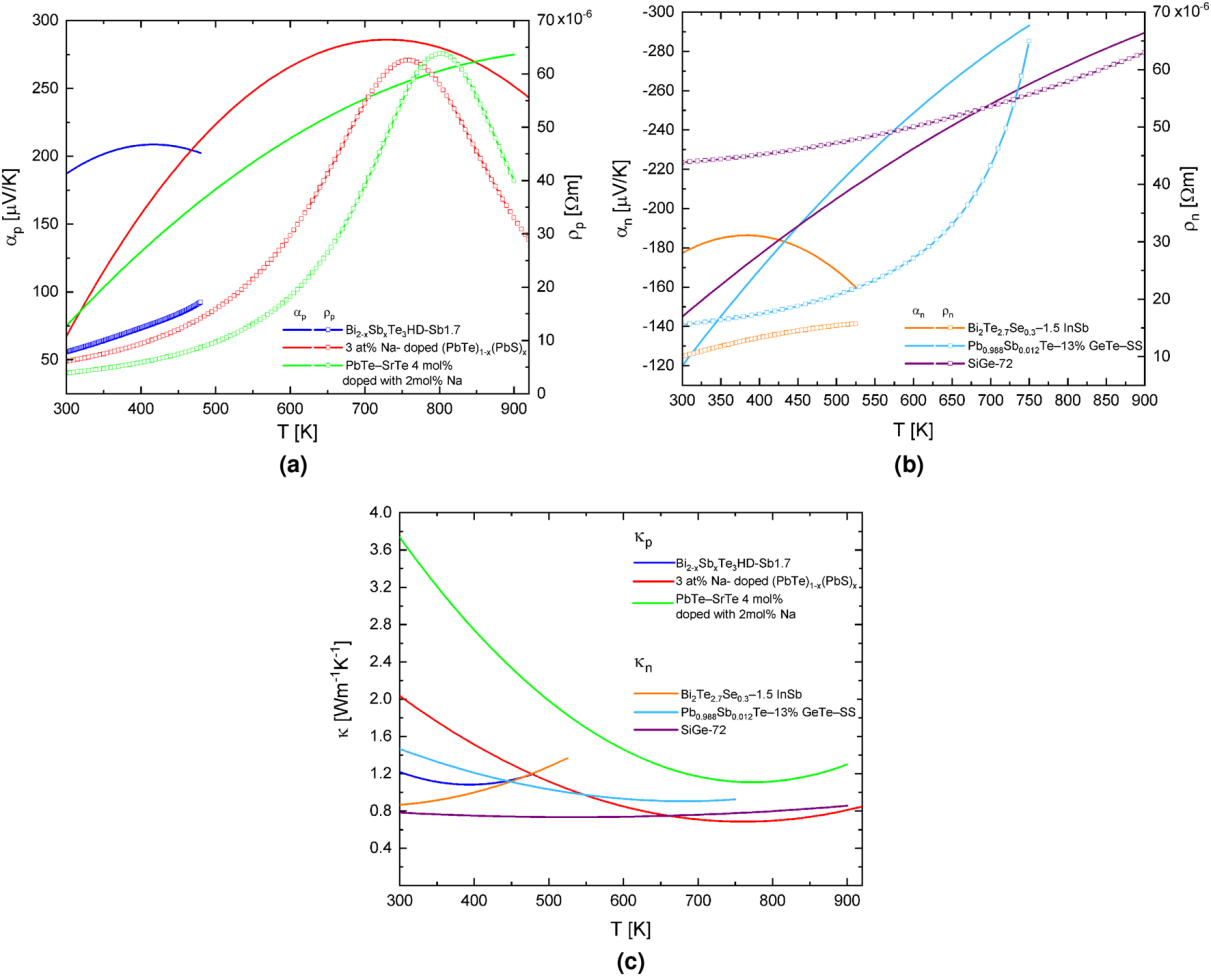
Temperature dependence of (**a**) Seebeck coefficient ($\alpha _p$) and resistivity ($\rho _p$) of the p-type material, (**b**) Seebeck coefficient ($\alpha _n$) and resistivity ($\rho _n$) of the n-type material and (**c**) Total thermal conductivity ($\kappa _p$, $\kappa _n$) for used TE materials.

In this paper, the temperature-dependent thermoelectric materials are used, $Bi_{2-x}Sb_xTe_3$ HD-Sb1.7, 3 at $\%$ Na-doped $(PbTe)_{1-x}(PbS)_x$, PbTe SrTe 4 mol $\%$ doped with 2 mol$\%$ Na for p-type segments and $Bi_2Te_{2.7}Se_{0.3}$-1.5InSb, $Pb_{0.988}Sb_{0.012}Te-13\%$ GeTe-SS, SiGe-72 for n-type segments. The materials and segmentation characteristics, such as length dimensions and material arrangement are shown in Table 1. It has been shown that these materials, normally used in energy harvesting applications, offer optimum efficiency. An approach of the thermoelectric properties values in function of the temperature is presented in Fig. 2 obtained from experimental data^6–8,22–24^.

**Table 1:** Dimensions of the segmented thermoelectric micro generator TEG.

Segment length	3p-2n	Segmentation material	2p-3n	Segmentation material
5.51 μm	$p_1$	PbTe SrTe 4 mol $\%$ doped with 2mol$\%$ Na	$n_1$	SiGe-72
2.91 μm	$p_2$	3 at $\%$ Na-doped $(PbTe)_{1-x}(PbS)_x$	$n_2$	$Pb_{0.988}Sb_{0.012}Te-13\%$ GeTe-SS
1.58 μm	$p_3$	$Bi_{2-x}Sb_xTe_3$ HD-Sb1.7	$n_3$	$Bi_2Te_{2.7}Se_{0.3}$-1.5InSb
7.72 μm	$n_1$		$p_1$	
2.28 μm	$n_2$		$p_2$	

The cross-sectional area for $\gamma =1$ is square $A_H=A_L=100$ μm^2^, for trapezoidal cases when $\gamma \ne 1$, the relationship between the smallest and largest cross-sectional area, it varies from $A_H=50$ μm^2^ and $A_L=100$ μm^2^ and inversely. The length of thermoelements p-type and n-type is 10 μm, but the length of each segment is shown in Table 1. The specific working temperature for each material is shown in Fig. 2, and the maximum temperature difference used in this paper is $\Delta T_ {max}$=600 K, with $T_c=300$ K.

M. Strasser et al.^25^ showed that to analyze the thermal behavior of thermoelectric generators at a microscale, standard mathematical models can predict the behavior of a micro thermoelectric generator. The simulations of the TEGs using the FEM-tool ANSYS revealed that the maximum power output was obtained with pure poly-Si to build the CMOS generators. This prediction of results by numerical simulation was verified in experimental tests. Therefore, and because we use the same software, the model proposed in this article can develop the multiphysics of energy conversion in two submodels, an electrical model and a thermal model using the finite element method.

### TEG: geometric shape factor, segmentation and load resistance

We consider two cases for the TEG: Symmetrical ($\gamma _p=\gamma _n$) and Asymmetrical ($\gamma _p \ne \gamma _n$) models. The change of the leg shape is as follows: firstly, there exists a trapezoidal shape when $\gamma =0.5$, then when $\gamma =1$, we have a rectangular shape, and finally, $\gamma =2$ an inverse trapezoidal shape is obtained.

#### Symmetrical

**Figure 3:**
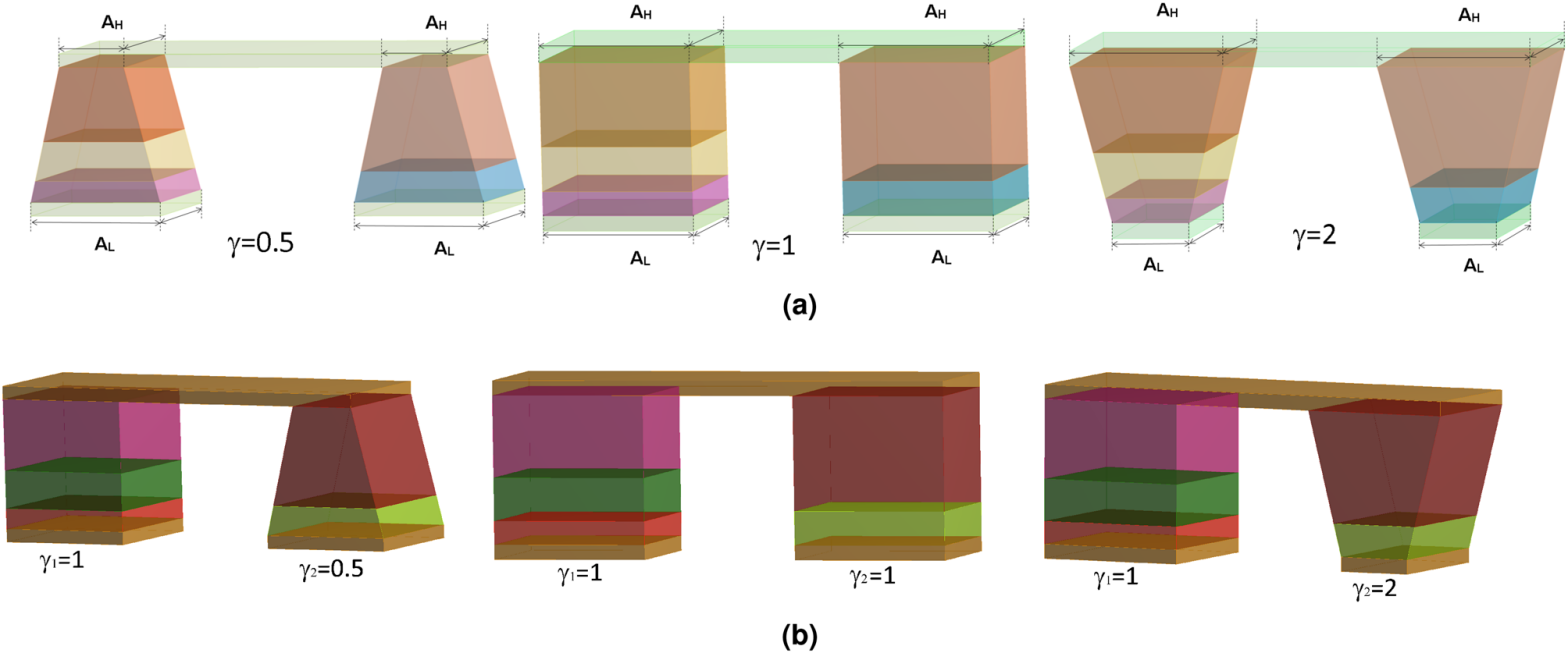
Evolution of the geometry of the elements in the TEG according to the geometric factor ($\gamma$) for (**a**) symmetric and (**b**) asymmetrical geometries.

In the symmetric model, the values of the geometric factor, $\gamma$, of the thermoelement p-type is equal to that of the n-type. The purpose of this work is to analyze all possible geometries that may exist between a trapezoidal geometry, in which cross-sectional areas are considered as $A_L>A_H$, and an inverse trapezoidal geometry, where $A_L<A_H$. Besides a rectangular geometry, $A_L=A_H$ is considered to include all geometries. The evolution of geometry of the element begins from the trapezoidal shape ($\gamma =0.5$). The area of the hot face increases until it reaches the rectangular shape ($\gamma =1$). Finally, the area of the cold face decreases until an inverse trapezoidal shape is obtained, ($\gamma =2$), as shown in Fig. 3a.

#### Asymmetrical

The asymmetrical models consider the asymmetric shapes, that is, the geometric shape of the p-type thermoelement is different from that of the n-type. In this case, the rectangular shape is kept constant for the p-type thermoelement, while for the n-type, the geometry of the thermoelement changes in the same way as previously described for the symmetric model shown in Fig. 3b.

#### Segmentation

Two different segmentation, using a different number of segments in the p-type and n-type thermoelements, is established to evaluate the effect of the configuration of the nanostructured materials and the geometric factor $\gamma$ on the performance of the TEG system. The proposed arrangements are, a) 3p-2n arrangement, and b) 2p-3n arrangement. Table 1 shows the geometric dimensions and the properties of the materials used in each model of the TEG, shown in Fig. 3a,b. The lengths of each segment are established according to dimensions previously proposed by Swanson et al.^26^ and also used by Lakeh et al.^27^. Cross-sectional areas, $A_H$ and $A_L$ vary according to geometric factor values, $\gamma$.

#### Resistance load

The thermoelements are electrically connected in series and thermally in parallel by copper electrodes and the load resistance, as shown in Fig. 1. We use an optimal load resistance of $R_L=5.83$ $\Omega$, considering the average values of the thermoelectric properties, as well as the geometric parameters of the rectangular shape.

Our novel approach considering these combined geometry effects allows us to analyze symmetrical and asymmetric geometries in an extensive temperature range, resulting in observable maximum peaks values in the performance of the TEG for specific $\gamma$ values. This significant enhancement in thermoelectric performance is also due to the incorporation of nanostructured materials. The analysis of the different system includes the most important physical parameters, which are previously used in the theory of optimal control in solid-state devices. Here we have carried out an analysis that allows us to solve the efficiency based on all the characteristic parameters and expand a methodology to identify the optimal design characteristics. Finally, we discuss a novel result in our analysis: new efficiency and output power surfaces, corresponding to different systems analyzed, are found as a function of the system operation, and important intersections between them occur at specific values of the geometric factor indicating equivalences in efficiency for different systems.

## Results and discussion

### Symmetrical models: efficiency and output power

**Figure 4:**
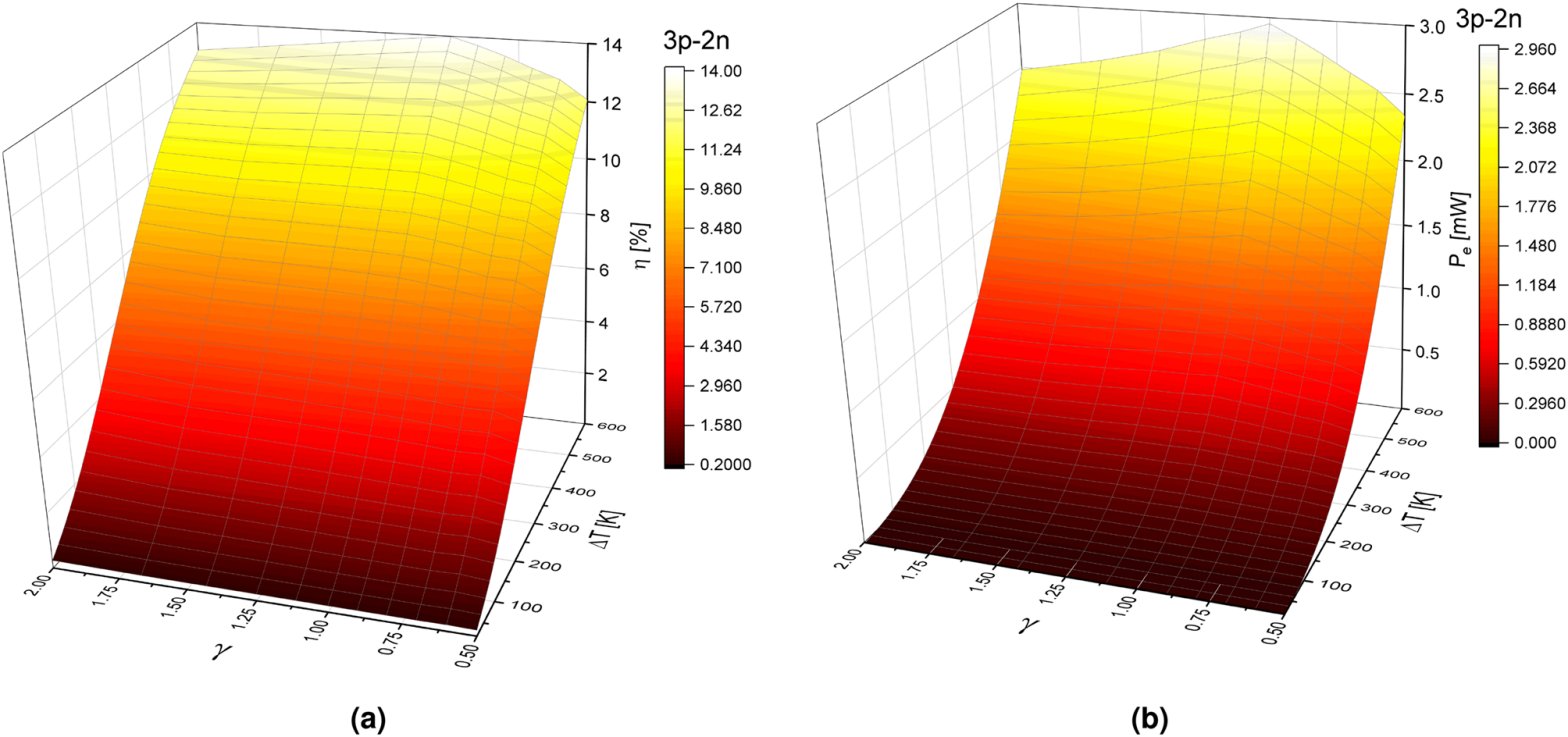
(**a**) Efficiency $(\eta )$ and (**b**) Output power $(P_e)$ in function of geometric factor $(\gamma )$ and temperature difference $(\Delta T)$ for a TEG in the 3p-2n model at $R_L=5.83$ $\Omega$.

**Figure 5:**
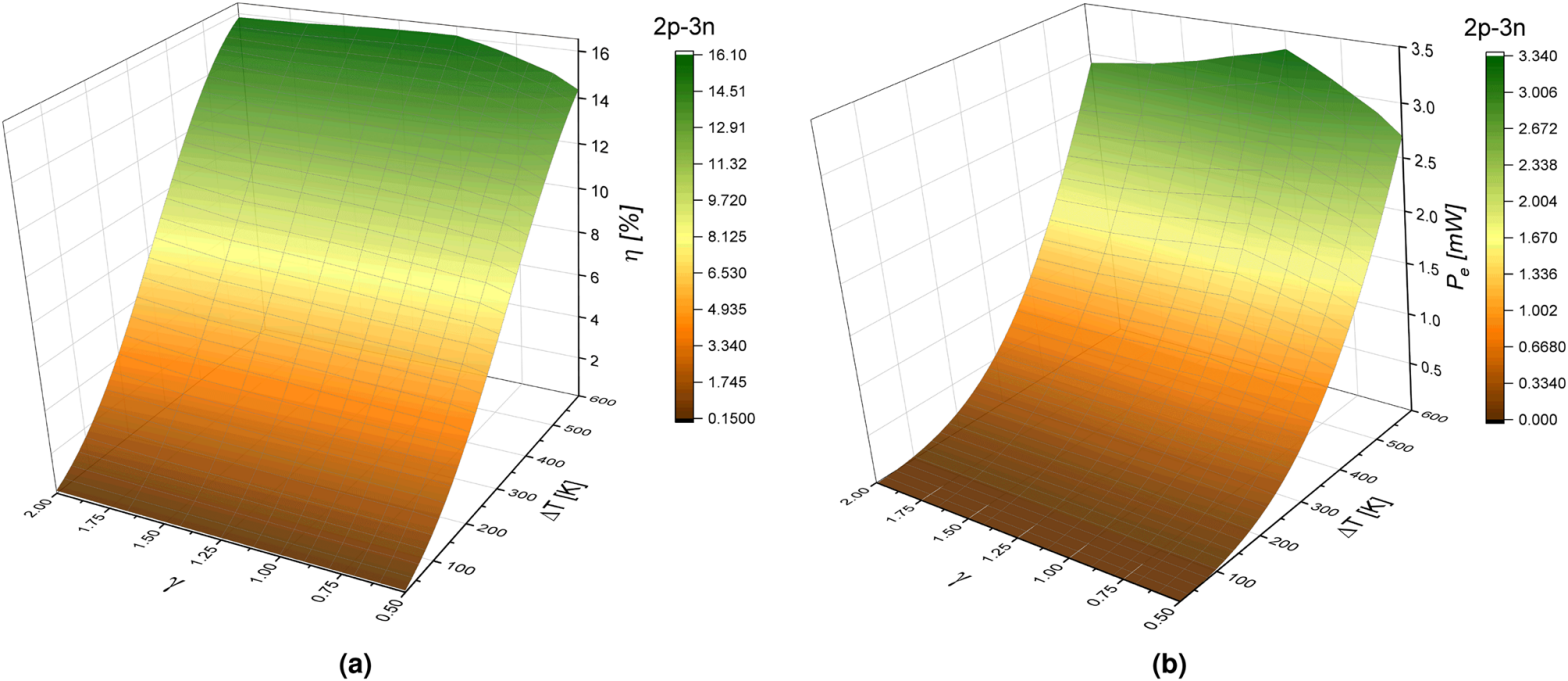
(**a**) Efficiency $(\eta )$ and (**b**) Output power $(P_e)$ in function of geometric factor $(\gamma )$ and temperature difference $(\Delta T)$ for a TEG in the 2p-3n model at $R_L=5.83$ $\Omega$.

The effect of geometry factor ($\gamma$) on the n-type and p-type elements, the configuration of materials, and the temperature difference $\Delta$T, on the performance of TEG is shown in Figs.  4 and 5 for arrangements 3p-2n and 2p-3n, respectively. Figure 4a,b show the efficiency and output power of the TEG as a function of the temperature difference and geometric factor $\gamma$ for arrangement 3p-2n. Figure 4a shows that efficiency increases with the increasing of $\Delta$T and for all possible values of the geometric ratio $\gamma$. Although efficiency increases for geometric factor values in the range of $0.5<\gamma <1$, the maximum efficiency $\eta =13.98 \%$ is reached when $\gamma =1$, for a rectangular shape, due to the load resistance value used.

It is observed that the efficiency decreases for values in the range of $1<\gamma <2$. For a value of $\gamma =0.5$, we get an efficiency of 12.07$\%$, and for $\gamma =2$, an efficiency of 13$\%$ is obtained. These results indicate that a system with cross-sectional areas such as $A_H>A_L$ offers higher efficiency values. As expected, results shown in Fig. 4b, for output power, are in accordance with efficiency results, i.e., the output power increases for values of a geometric factor in the range of $0.5<\gamma <1$, reaching the maximum output power of 2.95 mW with $\gamma =1$. The output power decreases for values in the range of $1<\gamma <2$. For values of $\gamma =0.5$ and $\gamma =2$ an output power of 2.34 mW and 2.48 mW are obtained, respectively.

Figure 5a,b show the efficiency and output power of the TEG as a function of the temperature difference, $\Delta$T, and the geometric factor $\gamma$ for the 2p-3n arrangement.

Figure 5a shows that efficiency increases for values in the range of $0.5<\gamma <1$ and decreases for values of $1<\gamma <2$ and again the maximum efficiency of 16.087$\%$ is obtained when $\gamma =1$. For values of $\gamma =0.5$ and $\gamma =2$ we get efficiencies values of 14.35$\%$ and 15.83$\%$, respectively. By comparing the difference between the systems 2p-3n and 3p-2n for maximum values ($\gamma =1$) and minimum values ($\gamma =0.5$) of the geometric factor effect on the efficiency, results show that in the 2p-3n system, the efficiency decreases by 11$\%$ compared with the 3p-2n system where it decreases 14$\%$.

Figure 5b shows that the output power increases for geometric factor values in the range of $0.5<\gamma <1$ and when $\gamma =1$ the maximum output power of 3.34 mW is obtained. The output power decreases for values in the range of $1<\gamma <2$. For a value of $\gamma =0.5$, an output power of 2.69 mW is obtained, and for $\gamma =2$, an output power of 2.91 mW is obtained. By comparing the difference between the systems 2p-3n and 3p-2n for maximum values ($\gamma =1$) and minimum values ($\gamma =0.5$) of the geometric factor effect on the output power, the results show that in the 2p-3n system, the output power decreases by 19$\%$ compared with the 3p-2n system where it reduces $21\%$.

These results show that for the 3p-2n system, under the same temperature conditions and the same load resistance, but considering a variable internal resistance, knowing that the internal resistance of the TEG depends on the thermoelectric properties of the material and the geometric parameters of the thermoelement, the output power and efficiency seems to be more affected than the 2p-3n system. This last statement is show in Figs. 4 and 5 where the higher system performance is obtained for higher temperature differences when $\gamma = 1$.

It has been shown that the output power and voltage-dependent on the geometry factor and thermoelectric properties. Our results are consistent with previous studies by Fan et al.^28^, who demonstrates that better performance in a TEG system is obtained with high temperatures differences using segmentation. In this paper, each nanostructured materials used works efficiently in a specific temperature range, as shown in Fig. 2. Therefore, knowing that each segment operates within an optimum temperature gradient, a proper material selection has been made for the best performance.

The thermoelectric properties for n-type materials can offer a better range of ZT values, for temperature ranges above 600 K, due to their thermoelectric properties improve performance at higher temperatures. It has been shown that the efficiency varies in function of the geometric factor $\gamma$ and that, under the same conditions of load resistance and temperature difference, changes in the efficiency for the 3p-2n system are smaller compared to the 2p-3n system. This implies that improper segmentation and geometry can also lead to a decrease in the performance of the TEG.

Therefore, according to the results obtained, the 2p-3n arrangement becomes a better option to obtain higher efficiency and output power. We can establish that a system with a cross-sectional area $A_H$ higher than $A_L$ is desired to obtain higher efficiency of the TEG. The performance of a device is reduced with the decrease in the area where the heat source is located, that is, when the heat transfer area is smaller on the hot side compared to the cross-sectional area on the cold side, the speed of heat transfer causes device performance to decrease.

### Asymmetrical models: efficiency and output power

**Figure 6:**
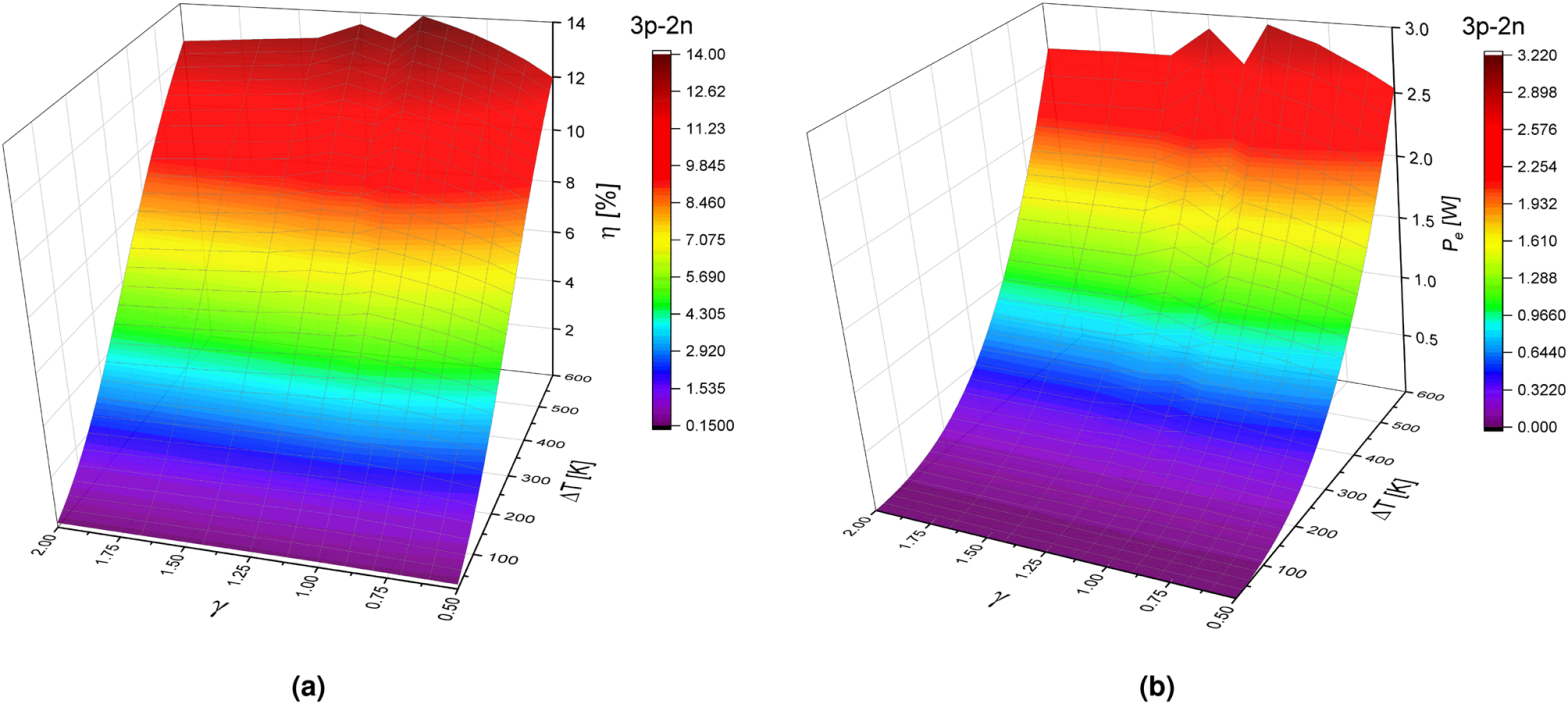
(**a**) Efficiency $(\eta )$ and (**b**) Output power $(P_e)$ in function of geometric factor $(\gamma )$ and temperature difference $(\Delta T)$ for a TEG in the 3p-2n segmentation at $R_L=5.83$ $\Omega$.

**Figure 7:**
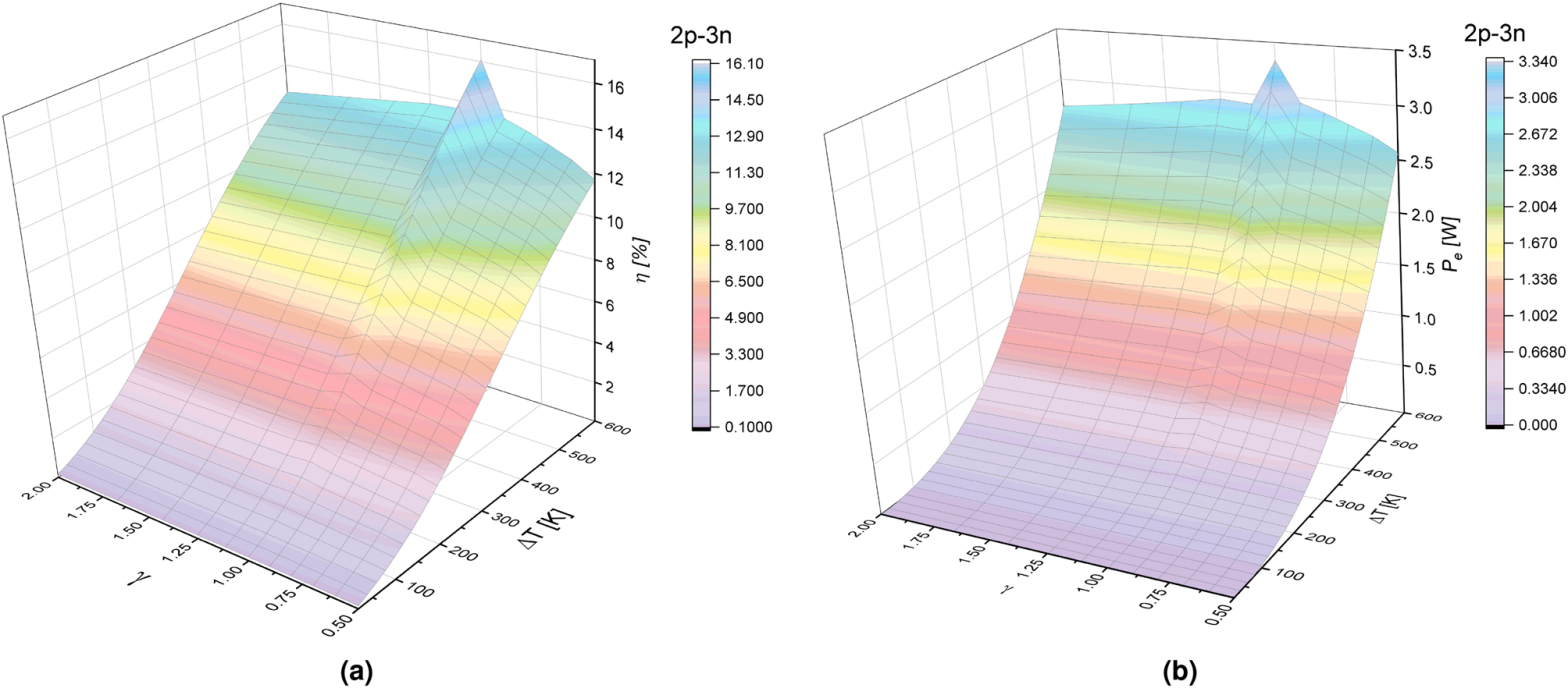
(**a**) Efficiency $(\eta )$ and (**b**) Output power $(P_e)$ in function of geometric factor $(\gamma )$ and temperature difference $(\Delta T)$ for a TEG in the 2p-3n model at $R_L=5.83$ $\Omega$.

The asymmetry and nanostructured materials effect on TEG performance is presented in Figs. 6 and 7 . The results from Fig. 6a show that the efficiency increases for values in the range of $0.5<\gamma <1$, where the maximum efficiency is $14\%$ with $\gamma =1$, which was expected because the load resistance was calculated according to with a rectangular system.

For the segmentation 3p-2n, slopes (or inflections) appear in the efficiency for critical values of the $\gamma$ geometric shape factor. After reaching maximum efficiency, with $\gamma =1$, the efficiency decreases in the range of $1<\gamma <1.11$, with an efficiency of minimum of 13.1$\%$, then efficiency increases in the range of $1.11<\gamma <1.25$ with an efficiency of 13.56 $\%$. Finally, the efficiency decreases for values in the range of $1<\gamma <2$.

The efficiency for $\gamma =0.5$ is $11.97\%$, while for a value of $\gamma =2$ it is $12.53\%$. This indicates that a system with a cross-sectional area $A_H$ higher than $A_L$ is desired for higher efficiency. In addition, in this case, the range of geometric factor values for $0.9<\gamma <1.25$ is desired to obtain better efficiencies values.

Figure 6b shows the output power as a function of the geometric factor and the temperature difference. The output power results also show maximum and minimum values in the same range of $\gamma$ values. The output power increases in the range of $0.5<\gamma <1$, the maximum output power is 2.95 mW at $\gamma =1$. After reaching the maximum output power, the output power decreases in the range of $1<\gamma <1.11$, with an output power of 2.62 mW, then increases again in the range of $1.11<\gamma <1.25$ with an output power of 2.88 mW. Finally, the output power decreases in the range of $1<\gamma <2$. For $\gamma =0.5$ the output power is 2.53 mW, $\gamma =2$ the output power is 2.62 mW.

Figure 7a shows the efficiency as a function of the temperature difference $(\Delta T)$ and the geometric shape factor ($\gamma$) for the asymmetric geometries in the 2p-3n segmentation. The efficiency increases in the range of $0.5<\gamma <1$, the maximum efficiency is of 16.087$\%$ with $\gamma =1$, then efficiency decreases in the range $1<\gamma <2$.

For $\gamma =0.5$ the efficiency is 11.7$\%$, and for $\gamma =2$ the efficiency is 12.63$\%$. In comparison with the 3p-2n segmentation, no inflections are observed with maximum and minimum values; it is only shown that the maximum efficiency is obtained when the geometric shape factor is the same in both thermoelements.

Figure 7b shows the output power as a function of the form factor ($\gamma$) and the temperature difference $(\Delta T)$, for the asymmetric geometries in the 2p-3n segmentation. The output power increases for values in the range of $0.5<\gamma <1$, the maximum output power is 3.34 mW at $\gamma =1$. The output power decreases in the range of $1<\gamma <2$. For $\gamma =0.5$ the output power is 2.56 mW, while that for $\gamma =2$ the output power is 2.72 mW.

In the 2p-3n segmentation, there are also critical values of the geometric shape factor $\gamma$, although these values are different in the 3p-2n segmentation. In the range of values of $0.9<\gamma <1.11$, the efficiency and output power values increase dramatically with small changes of $\gamma$. It determines the relation of areas that must be considered to achieve an optimization of TEG. For asymmetric geometries systems, the 2p-3n system turns out to be better than the 3p-2n system. It is showed that a system with a higher cross-sectional, $A_H > A_L$, is desired to obtain higher efficiency and output power.

Asymmetric geometric systems show lower performance than conventional segmented thermoelectric devices—this due to the load resistance. In the asymmetric systems, since the internal resistance of the trapezoidal shape is different from the rectangular shape, the heat flux is affected. In trapezoidal shapes, the heat transfer rate is lower in small cross-sectional areas. In contrast, the heat flux is homogeneous for rectangular shapes. The maximum efficiency and power values for the symmetric and asymmetric models in the two segmentations 3p-2n and 2p-3n are shown in the Table 2.

**Table 2:** Comparison of maximum efficiency and output power values at $\Delta$T = 600 K and $R_L=5.83 \Omega$ for symmetric and asymmetric models.

Parameter	Trapezoidal shape ($\gamma$ = 0.5)		Rectangular shape ($\gamma$=1)		Inverted trapezoidal shape ($\gamma =2$)	
Segmentation	$\eta _{max}$ (%)	$P_{out}$(mW)	$\eta _{max}$ (%)	$P_{out}$ (mW)	$\eta _{max}$(%)	$P_{out}$ (mW)
**Symmetrical models**						
3p-2n	12.07	2.34	13.98	2.95	13	2.48
2p-3n	14.35	2.69	16.087	3.34	15.83	2.91
**Asymmetrical models**						
3p-2n	11.97	2.53	14	2.95	12.53	2.62
2p-3n	11.7	2.56	16.087	3.34	12.63	2.72

Our work analyzes the performance of the segmented TEG, for different cases under the same operating conditions with a wide range of possibilities, due to the variable value of $R_{int}$, since its in the function of the temperature difference and geometric shape factor, which allows improving the overall performance of the thermoelectric device.

The effect of asymmetric segmented thermoelements on the performance of a thermoelectric generator has shown a significant improvement compared to standard rectangular thermoelements. Our results show the performance behavior for the symmetric and asymmetric segmented system. For asymmetric shapes, by establishing the same operating conditions to standard thermoelements but considering the optimal load resistance ratio and the internal resistance, which both differ from the standard form, power output and efficiency show peaks of maximum and minimum performance depending on the geometric form factor $\gamma$. Therefore, an unstable performance behavior is shown compared to a symmetric system, which will always have its maximum performance when considering the standard form, that is, $\gamma =1$, and considering a constant load resistance.

### Effect of load resistance

In previous work, the investigation of the thermoelement geometric shape together with the load resistance showed that the performance of a thermoelectric device depends directly on the load resistance and the internal resistance of the TEG, due to a decrease in the thermal and electrical conductivity, which in turn produces a reduction in the temperature difference Besides, previous works have shown that it is possible to determine the angle of inclination and the shape of the thermoelements in order to improve the TEG performance. However, in most studies, an optimized constant load resistance calculated according to average values is taken into account. A constant load resistance limits the scope of the maximum performance of the TEG. The results shown in this work reveal the real effect of the geometric shape and the temperature-dependent thermoelectric properties on the performance of the TEG. Then, to obtain the maximum performance, the load resistance must be chosen in relation to the internal resistance based on the geometric shape factor and the temperature-dependent thermoelectric properties of the nanostructured materials.

**Figure 8:**
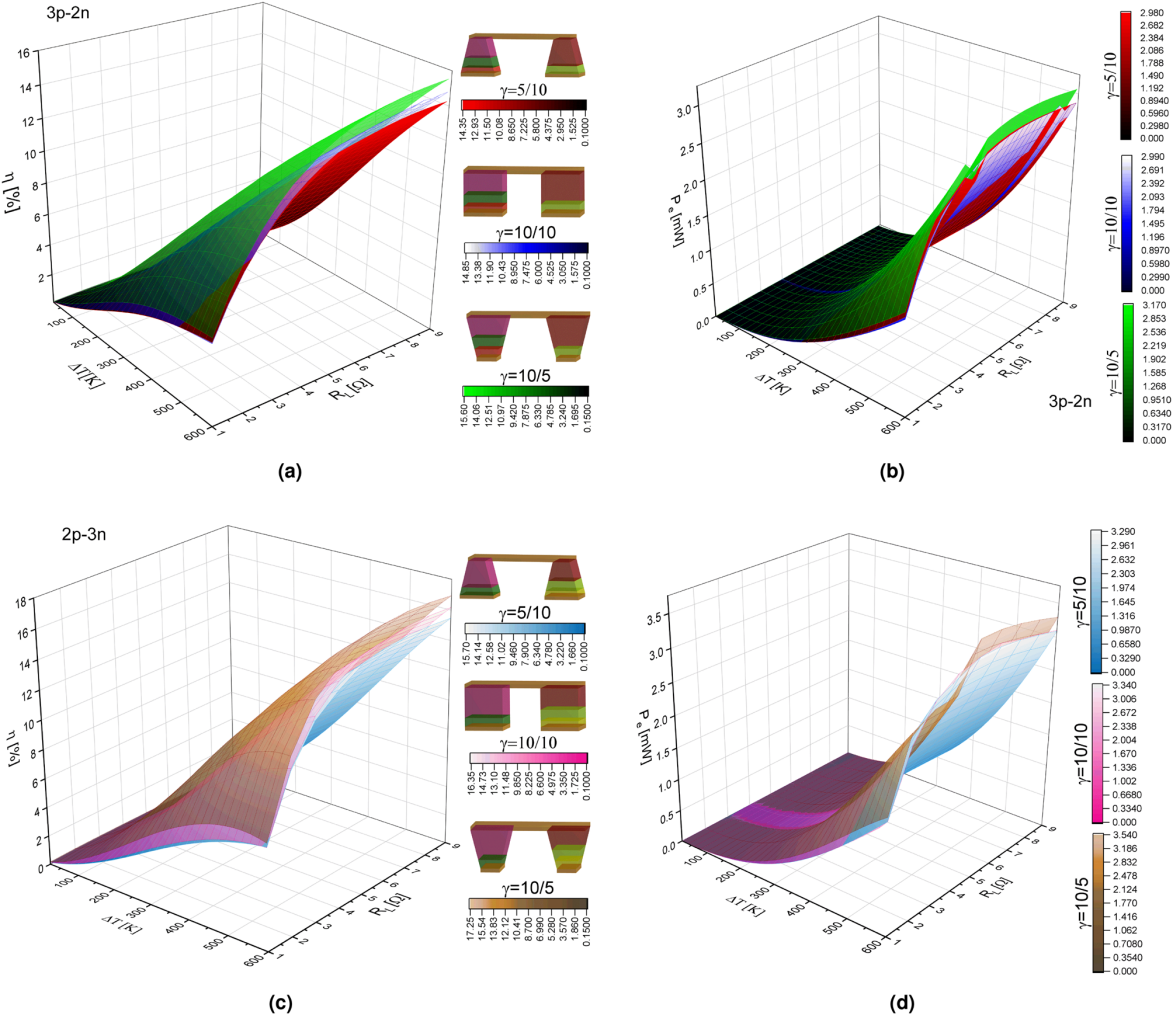
(**a**) Efficiency $(\eta )$ and (**b**) Output power $(P_e)$ in function of load resistance $(R_L)$ and temperature difference $(\Delta T)$ for a TEG, geometric factor $\gamma =5/10, 10/10, 10/5$ in the 3p-2n model and (**c**) Efficiency $(\eta )$ and (**d**) Output power $(P_e)$ for 2p-3n model.

Figure 8a–d show the efficiency and output power of the TEG as a function of the temperature difference, $\Delta$T, and the load resistance, $R_L$, for the 3p-2n and 2p-3n arrangement considering three main geometric shapes, $\gamma =0.5$ (trapezoidal shape), $\gamma =1$ (rectangular shape) and $\gamma =2$ (inverted trapezoidal shape).

It is observed that the efficiency increases with $R_L$ and $\Delta$T values. It should be noted that in Fig. 4a, the best performance is obtained with the rectangular geometry. Nevertheless, considering different load resistance values, the inverted trapezoidal shape reaches maximum efficiency values, higher than the rectangular shape.

Figure 8c shows the efficiency as a function of the load resistance and the temperature difference for the 2p-3n array. It is observed that the efficiency increases as $R_L$ and $\Delta$T increase. The increase in efficiency in the inverted trapezoidal shape, where a maximum efficiency of 17.25$\%$ is obtained, is more notable compared to Fig. 8a, being this efficiency the maximum obtained from all the analyzed systems. The maximum efficiency of the TEG is obtained for $\gamma =10/5$ and a load resistance of $R_L=7 \Omega$ in the 2p-3n arrangement.

Figure 8b,d show the output power as a function of the load resistance and the temperature difference for the three main geometric shapes for the 3p-2n and 2p-3n arrangement. Similar results are observed between both arrangements; however, notable differences are shown in the inflections areas. Maximum efficiency, $\eta _{max}=17.23527\%$, is obtained for the geometric value of $\gamma =2$ (inverted trapezoidal geometry) with a load resistance value of $R_L=7 \Omega$, as we can see in Fig. 8c for the 2p-3n arrangement, compared to a rectangular shape, $\eta =16.087\%$, for a value of $\gamma =1$ with a load resistance $R_L=5.83$ $\Omega$, as we see in the Fig. 5a. These results are consistent with Karana et al.^29^, which shows that an asymmetrical geometric factor has a higher performance when the cross-sectional area of the thermoelement is larger on the hot face, in addition to having a high load resistance ratio. The results are shown in Fig. 8, for maximum output power and efficiency with maximum temperature difference $\Delta$T=600 K and different load resistances, are shown in the Table 3.

**Table 3:** Comparison of maximum values of efficiency and output power at $\Delta$T=600 K.

Parameter	Trapezoidal shape ($\gamma$=0.5)		Rectangular shape ($\gamma$=1)		Inverted trapezoidal shape ($\gamma$=2)	
Arrangement	$\eta _{max}$ (%)	$P_{out}$(mW)	$\eta _{max}$ (%)	$P_{out}$ (mW)	$\eta _{max}$(%)	$P_{out}$ (mW)
3p-2n	14.3158	2.9780	14.8432	2.98225	15.58796	3.16254
	$R_L=8.89\, \Omega$	$R_L=6.2265\, \Omega$	$R_L=8.89\, \Omega$	$R_L=8.0055\, \Omega$	$R_L=8.89\, \Omega$	$R_L=6.2265\, \Omega$
2p-3n	15.67507	3.28645	16.30792	3.33378	17.23527	3.5368
	$R_L=7\, \Omega$	$R_L=5\, \Omega$	$R_L=7\, \Omega$	$R_L=6\, \Omega$	$R_L=7\, \Omega$	$R_L=5\, \Omega$

The global temperature difference defines the operating range for each material, which implies that the internal resistance of the TEG depends on $\Delta$T. Each segment operates with a temperature difference that may or may not be the optimum for the material. This fact plays a crucial role since the internal resistance and geometric factor impose the efficiency of the TEG due to the nanostructured nature of the materials in combination with the relationship of the geometric factors.

The geometric factor effect on TEG performance and temperature profile is minimum in a thermoelement with homogeneous cross-sectional area and constant volume. Therefore, by comparing rectangular thermoelements to cylindrical and octagonal thermoelements, their performance is approximately equal, this fact is because the internal resistance of the generator is similar regardless of the geometric factor^9,30^.

The geometric factor of the element did not have any significant influence on the maximum power in some specific load resistance values. This is because the maximum power was calculated using different temperature differences, and the internal electrical resistance is, in turn, calculated in function of the geometry of the elements. Considering the same temperature difference between the internal resistances, we can generate the same output power, and the surface differences are small enough not to distinguish operation condition patterns. However, our results show that the efficiency and out power increases almost linearly with the temperature and the geometric factor: increasing temperature difference increases the hot side temperature. Consequently, at a higher temperature, higher efficiency is obtained for improving the conversion efficiency of the device. This matter because, with making a thermal difference at both sides of the n-type semiconductor, which has a negative Seebeck coefficient, electron flux will release toward the cold side that has less level of energy. Consequently, the negative electric potential will emerge on this side, and in the p-type arm which has positive Seebeck coefficient, holes will treat unlike of electrons in n-type arm.

The trapezoidal shapes have the same volume as the rectangular shape. However, they have a cross-sectional area that is not homogeneous along the thermoelement, causing a different internal resistance of the TEG compared to the rectangular one; this effect modifies the optimal ratio between load resistance and internal resistance with a significant impact on performance. Maximum efficiency and output power can be obtained when the load resistance is equal to the internal resistance of the TEG. However, when considering temperature-dependent materials, the internal resistance varies with the temperature difference. Therefore, the external load resistance varies until the maximum performance of the TEG is obtained, which represents the corresponding load condition for each system.

In this study, we numerically simulate both by controlling the external resistance. The output power becomes smaller when is smaller, i.e. $\gamma =0.5$ to $\gamma =1$. This is because the internal electrical resistance increases as $\gamma$ decreases. According to Eq. (4), the available power becomes smaller when the electrical resistance increases but increases with the load resistance. In these results, we show the tendency for the maximum power to increase with increasing $\gamma$; it is found that surface patterns of output power intersect with each other in the different elements analyzed. It was revealed that the modification of element shape has a significant influence on the maximum power and the output voltage is strongly dependent on the external load resistance. Finally, if the load resistance and temperature difference are in the maximum values, output power would increase considerably, as shown in Fig. 8d for the $\gamma =10/5$ case when $A_H > A_L$.

In this work it has been shown that the geometric factor together with the load resistance plays an important role on TEG performance and its impact on performance is even more significant when the cross-sectional area is not homogeneous. The results show that an incorrect selection of load resistance for the trapezoidal shape causes a performance below the rectangular shape as shown in the Figs. 4a and 5 a.

However, with the inverted trapezoidal shape and the optimal load resistance, maximum performance is obtained, above the rectangular shape, as shown in the Fig. 8a,c. This fact can be explained with the temperature distribution; that is, the inverted trapezoidal shape can improve the temperature distribution of its thermoelement, having a favorable effect on the generation of Seebeck, showing an increase in efficiency and output power of TEG.

It is well known that experimental results differ from simulations results since small variations of size can produce changes in performance. Previously investigation of TEG’s attempt to identify the parameters causing these differences. If a constant temperature difference is assumed, mathematical solutions are easy and suitable; however, TE modules do not work in ideal parameters. Thereby, it is possible to estimate surfaces of efficiency and output power in this situation and to establish equivalence between different structures. Understanding the physical aspects investigated in this paper in conjunction with the thermoelectric properties of heterogeneous element structures is critically important for future design and advancement in solid-state devices. Temperature-dependent properties material is used to ensure result accuracy, as is presented in Fig. 2. Thus, with the presented results, we propose a novel design guide for segmented thermoelectric in the function of operation conditions:1. For segmented systems, a 2p-3n segmentation must be chosen. 2. Symmetrical model takes precedence over asymmetrical models. 3. A geometric factor of $\gamma =10/5$, i.e. trapezoidal elements, should be used. 4. In general, the hot and cold areas must be $A_H > A_L$. 5. The materials $n_1$ and $p_1$ must have a better Seebeck coefficient and withstand high temperatures because they are in contact with the hot surface. 6. Rectangular and trapezoidal systems can be used under the same operating conditions, depending on $\Delta T$ and $R_L$, and to make the best choice we must, consider the ease of manufacture.

With the proper selection of design parameters, we can establish a direct relationship between the systems studied under various operating conditions, and thus, expand the range of application of these devices by not being limited by their geometric shape.

A combination of electrochemical deposition techniques and integrated circuit technology have been developed to manufacture high-performance microdevices. It has been shown that electrodeposition both n-type and p-type thick $Bi_2Te_3$ alloy films with a thickness of 10–60 μm from aqueous solutions can achieve transport properties similar to those of bulk materials^31^. The fabrication of TEG’s has also been shown using high ZT nanostructured bulk (nanobulk) half- Heusler alloys using a highly scalable process^21,32^.

In this work, we report the behavior of various high ZT nanostructured thermoelectric materials, which for the purposes of waste heat energy harvesting applications, could be used for nanobulk applications. However, in future work, the more extensive analysis on the effect of the size of nanostructured materials for applications such as thin and thin films, nanowires among other,s will be investigated.

## Conclusion

Traditionally, the analysis of thermoelectric device optimization methods is limit by a focus on a specific objective. In this paper, a new multi-objective study for a TEG optimization model has been described according to geometric factor, segmentation, load resistance, and temperature-dependent nanostructured material properties. The following conclusions are obtained from the theoretical performance analysis of a TEG with segmented symmetric and asymmetric thermoelements and nanostructured materials.

In the 2p-3n symmetric system, when the load resistance is established according to average properties material and $\gamma \ne 1$, results in the figures shows a slope of decrease in efficiency and output power. However, the rate of change in the performance decrease is lower in the 2p-3n system compared to the 3p-2n system.

The effect of asymmetrical shape in the thermoelements on performance shows slight inflections, for a specific value ranges of $\gamma$, in the performance of efficiency and output power graphics. In the 3p-2n system, maximum and minimum efficiency values are obtained for the range values of $1<\gamma <1.11$ while in the 2p-3n system, for the range values of $0.9<\gamma <1.11$ the efficiency and output power increases rapidly to a maximums peak values.

Compared with previous work, the temperature dependence of the nanostructured materials and the geometric form factor are considered in this work, which results in changes in the internal resistance for all temperature gradients considered. Still, the value of the load resistance remains constant. With these considerations, new slopes (or inflections) appear in the efficiency for critical values of $\gamma$, demonstrating that the relationship $m=R_L/R_{int}$ must be adjusted according to the required operating condition in order to reach an adequate optimization.

However, when modifying the load resistance, it is shown that the inverted trapezoidal geometry obtains an increase in efficiency in 7$\%$ higher than the rectangular geometry. This fact establishes the selection of different linked optimization criteria according to geometric factors, load resistance, and internal electrical resistance, which allows obtaining a better performance of the TEG with an adequate selection of the load resistance.
